# WJES: how to review a clinical paper

**DOI:** 10.1186/1749-7922-4-8

**Published:** 2009-02-10

**Authors:** Kaoru Koike, Luca Ansaloni, Fausto Catena, Ernest E Moore

**Affiliations:** 1 Emergency Surgery Department, St Orsola-Malpighi University Hospital, Bologna, Italy; 2 Department of Primary Care & Emergency Medicine, Kyoto University Graduate School of Medicine, Kyoto, Japan

## Editorial

World Journal of Emergency Surgery (WJES) was started to encompass all aspects of clinical and basic research studies related to emergency surgery and its allied subjects. Emergency surgery is a multidisciplinary super-specialty involving all surgical specialties and all emergency medicine specialties. Emergency surgery is divided into traumatic and non-traumatic emergency surgery. WJES accepts the following types of articles: research, case reports, reviews, book reviews, commentaries, letters to the editor, methodology articles, and study protocol.

Table 1 shows how many papers have been published in each type of articles during the first 2 years, from the launch of this journal until July 2008. The total number of publication is 96. The acceptance rate is 46%. The number of case reports is the most, 40 papers and 42% of all publication. The research articles are 24 (25%) and reviews are 23 (23%). The acceptance rate of case reports is 35%, which was 15% at the time of December 2006 [1].

**Table 1:** Number of papers published in WJES

Article Type	accepted/submitted	acceptance rate (%)
Case report	40/115	35
Research article	24/52	46
Review	23/28	82
Editorial	6/6	100
Letters to the Editor	1/1	100
Methodology	1/1	100
Study protocol	1/4	25
Book review	0/0	-
Commentary	0/1	0
		
Total	96/208	46

March 2006–July 2008

Table 2 shows which countries these WJES papers were submitted from. It appears that papers are still sent from a relatively limited number of countries.

**Table 2:** Countries where published papers in WJES came from

	Research	Case	Review	Editorial	Letter	Method	Protocol	Total
UK	5	17	5					27
Italy	3	5	3	5			1	17
USA	4	3	7					14
Turkey	5	2	2					9
India	2	3	1		1			7
Israel		1	2			1		4
Ireland	1	3						4
New Zealand	2	1						3
Brazil		1	1					2
Germany		1		1				2
Finland	1	1						2
France	1							1
Croatia		1						1
Singapore		1						1
Netherland			1					1
Japan			1					1
								
Total	24	40	23	6	1	1	1	96

March 2006–July 2008

Table 3 indicates what kinds of papers are included in each type of articles. Research articles are usually classified into prospective, retrospective, or observational studies. The detail of each will be discussed later. When we look into the research articles in WJES, the rate of traumatic paper was 42% and the number of basic paper was 3. Two of the basic research articles dealt with healing of colonic anastomosis in rats [2,3] and the other one demonstrated data obtained from a mathematical model [4]. Among clinical research papers one was prospective and the others were retrospective [5].

**Table 3:** Classification of published papers in WJES

	basic	clinical		traumatic	non-traumatic
Research article	3 (13%)	21 (87%)		10 (42%)	14 (58%)
Case report	0 (0%)	40 (100%)		8 (20%)	32 (80%)
Review	0 (0%)	23 (100%)		10 (43%)	13 (57%)

March 2006–July 2008

Publishable case reports should meet one of the categories: 1) the first report of a new entity, 2) additional examples that establish an entity from an isolated observation, 3) adverse events, 4) a remarkably well-documented example of educational value, or 5) other [1]. When we look at case reports in WJES, 80% of them were non-traumatic. At this moment emergency surgeons appear to select WJES for the place sending non-traumatic emergency case reports in.

Taken together we will keep welcoming retrospective papers and case reports but pay attention to the quality control. When World Society of Emergency Surgery (WSES) planned and performed sophisticated clinical studies and guidelines, the value of WJES will certainly raise. We are looking forward to the 1^st ^congress WSES held in 2010 at Bologna, Italy.

## Authors' contributions

All authors contributed equally to this work
